# From Old to Bold: Advancing microRNA Studies in Sudden Cardiac Death Through Molecular Analysis of FFPE Heart Tissue

**DOI:** 10.3390/genes17070819

**Published:** 2026-07-17

**Authors:** Alessia Bernini Di Michele, Chiara Turchi, Mauro Pesaresi

**Affiliations:** Section of Legal Medicine, Department of Biomedical Sciences and Public Health, Polytechnic University of Marche, Via Tronto, 60126 Ancona, Italy; a.bernini@pm.univpm.it (A.B.D.M.); m.pesaresi@univpm.it (M.P.)

**Keywords:** sudden cardiac death, molecular autopsy, miRNA, FFPE tissue

## Abstract

Background/Objectives: Sudden cardiac death (SCD) is a natural death of cardiac origin, accounting for an estimated 6–9 million deaths worldwide each year and representing a major public health challenge. Despite its clinical and forensic relevance, the molecular investigation of SCD remains limited. Peripheral blood or fresh tissue, the preferred specimens for post-mortem genetic testing, are not always available, and DNA extracted from formalin-fixed paraffin-embedded (FFPE) tissues is often suboptimal for conventional genetic analyses. This review evaluates the potential of archived FFPE cardiac tissue as a source for microRNA (miRNA) analysis in molecular autopsy. Methods: A narrative review was conducted by collecting studies investigating miRNA expression in FFPE cardiac tissue relevant to SCD. Ten studies met the inclusion criteria and were critically analyzed. Results: Although the available evidence remains limited, recent studies have identified several differentially expressed miRNAs associated with cardiac diseases relevant to SCD. Owing to their small size and remarkable stability, miRNAs remain detectable in FFPE tissues despite fixation and long-term storage, making them attractive molecular biomarkers. While most available studies were conducted in clinical rather than forensic settings, they demonstrate the feasibility and analytical robustness of miRNA profiling in archived FFPE cardiac specimens. Conclusions: This review underlines the importance of reconsidering archived FFPE tissues not merely as historical or morphological resources, but as promising matrices for molecular autopsy of SCD supporting the identification and validation of novel molecular biomarkers.

## 1. Introduction

Sudden cardiac death (SCD) is defined as a natural, nonviolent death presumed to be of a cardiac cause that occurs within 1 h of the onset of cardiac symptoms or 24 h of last being seen healthy and alive [1,2]. SCD has always been a widespread phenomenon among the population, with an estimated global burden of approximately 6–9 million deaths annually, accounting for 10–15% of all deaths globally. Risk rises exponentially with age and occurring more frequently in men than in women [3,4,5].

Cardiac pathologists and coroners are tasked with determining the cause and mechanism of SCD, whether in a hospital setting or within forensic investigations.

In forensic medicine, post-mortem genetic testing, known as molecular autopsy, represents a valuable complementary tool for identifying the cause of SCD in cases that remain unexplained after a comprehensive conventional forensic autopsy (negative autopsy) [6]. Post-mortem genetic analysis is recommended in most cases but is currently not always performed. Post-mortem testing can identify gene variants in around one-third of cases. In SCD cases, it has been shown that combining post-mortem testing and family investigation of first-degree relatives can lead to a diagnostic yield of 40% [7]. Nonetheless, tissue and blood retention for DNA extraction would be crucial in allowing post-mortem genetic analysis for heritable cardiovascular disorders. Unfortunately, optimal specimens—such as peripheral blood—are often unavailable because they are not routinely preserved during autopsy. Formalin-fixed paraffin-embedded (FFPE) tissue is ubiquitously collected at autopsy, but the poor quality of the DNA extract from these specimens certainly limits the use of traditional sequencing methods. Fixation in formalin-based solutions, followed by paraffin embedding to produce FFPE tissue blocks, represents the gold-standard approach for preserving human tissues for diagnostic purposes. Processing material through this method has several advantages, ranging from reducing risks of infectious agents potentially present in the fresh material to ensuring preservation of the structural integrity of the tissue. Embedding in paraffin wax allows for the preparation of thin sections, enabling detailed examination of tissue architecture with basic stains such as hematoxylin and eosin, which help distinguish the various cellular components. A major limitation of FFPE tissue for molecular analyses is the introduction of formalin-induced DNA damage, which may generate sequencing artefacts. During next-generation sequencing (NGS), these artefactual changes can be mistakenly interpreted as genuine genetic variants, despite not being present in the original biological sample. Consequently, distinguishing true pathogenic mutations from fixation-related artefacts is essential to ensuring the reliability of genetic analyses performed on FFPE-derived DNA. Several methodological strategies have been proposed to minimize these errors, including the use of DNA polymerases with reduced capacity to bypass damaged DNA templates, thereby decreasing the likelihood of incorporating fixation-induced artefacts into sequencing results. Steiert et al. [8] demonstrated the importance of critical parameters that most affect sequencing results by generating DNA sequences from older FFPE samples and comparing this to DNA from fresh-frozen tissue. Although some problems can be managed, others cannot be fully controlled. The pivotal step in any diagnostic procedure is the preparation and isolation of high-quality starting material. This requires the careful optimization of both sample collection and preservation methods. Since no universally accepted standard exists for tissue fixation, the wide variability in preservation techniques hinders subsequent preparative and diagnostic processes. Moreover, most diagnostic laboratories receive material from multiple clinical centers, and even minor differences in standard fixation protocols can lead to substantial variations in the quality and yield of extracted DNA. The most used method for tissue preservation in medical applications is formaldehyde fixation followed by paraffin embedding. While this approach effectively preserves tissue architecture, cell morphology, and intracellular components (proteins, carbohydrates, etc.), prolonged formalin fixation also induces crosslinking between proteins and nucleic acids and generates random breaks in nucleotide sequences. Although several strategies exist to mitigate these issues, molecular analysis on FFPE tissue samples remains highly challenging. FFPE-specific artefacts—false positives observed in FFPE-derived DNA—can also occur and may be misinterpreted as true variants, independent of the actual individual-specific alterations. Recent studies [9] have shown that the diagnostic yield of molecular autopsy may be reduced when only archived FFPE tissues are available because DNA quality can be adversely affected by fixation, storage, and contamination. These observations further justify the search for complementary molecular biomarkers that remain analytically reliable in FFPE specimens.

In recent years, however, attention has shifted toward alternative biomarkers, such as microRNAs (miRNAs). miRNAs are small non-coding RNA of approximately 20–22 nucleotides, inherently stable, very robust and resistant to severe changes in pH or temperature, and their expression levels remain consistent when comparing fresh-frozen tissue to FFPE tissue [10,11,12]. On the contrary, mRNA undergoes greater degradation, given its longer conformation. Moreover, studies on specimens stored for 12–20 years in FFPE blocks demonstrated that miRNAs remain stable for long periods [13,14]. For these reasons, miRNAs can be considered optimal biomarkers for post-mortem analyses on FFPE tissues.

One of the main contexts in which FFPE tissues are used for molecular analyses is when no other biological matrix is available. In addition, this type of specimen can be stored at room temperature for years. In forensic settings, FFPE blocks are often preserved for long periods and, when needed, high-quality analytical techniques can still be applied even years after their initial preparation. The most frequent forensic applications involve FFPE heart tissue, particularly in cases of SCD.

The purpose of this review is to emphasize miRNAs as new biomarkers that may be helpful in genetic analysis for SCD, particularly when using complex biological matrices such as FFPE. To this end, we have highlighted features and potential advantages of using miRNAs in molecular analysis of SCD. This review showed studies on miRNA analysis from FFPE heart tissue with the aim of being able to also use these findings for forensic purposes, in the field of sudden cardiac death. Given the limited number of available studies specifically addressing miRNA analysis in FFPE cardiac tissue for sudden cardiac death investigations, this review aims to provide a comprehensive narrative overview of the current evidence, highlighting methodological approaches, principal findings, existing limitations, and future perspectives for forensic applications.

## 2. Literature Search and Study Selection

A comprehensive literature search was conducted to identify original studies investigating miRNA analysis in FFPE cardiac tissue. Given the narrative and critical nature of this review, the search strategy was designed to explore miRNA stability in FFPE tissues, their role on cardiac pathologies and SCD, methodological advances and miRNA expression profile. The search was performed using PubMed https://pubmed.ncbi.nlm.nih.gov/ (accessed on 21 may 2026) and Scopus https://www.scopus.com/pages/home#basic (accessed on 21 May 2026) databases. The search strategy combined the following keywords and Boolean operators: (“FFPE” OR “formalin-fixed”) AND (“heart” OR “cardiac” OR “myocardial”) AND (“miRNA” OR “microRNA”). We included peer-reviewed original research articles, longitudinal studies, pilot trials, and forensic case series published in English that provided primary quantitative or qualitative data on myocardial miRNA stability, degradation kinetics, or differential expression patterns.

Overall, ten studies fulfilled the inclusion criteria and were considered relevant to the objectives of this narrative review.

A comprehensive summary of the studies highlighting study design, patient populations, analytical methodologies, and principal findings of all included studies is provided in Supplementary Table S1.

## 3. miRNA Stability in FFPE Tissue

One of the earliest studies investigating the stability of mRNAs in FFPE tissue was conducted by Li in 2007 [15]. This study compared the stability of 160 miRNAs in FFPE samples with that in fresh-frozen tissue through a qRT-PCR, highlighting the potential of these molecules for genetic analysis. Researchers first analyzed miRNA expression in thawed tissue samples and subsequently compared the results with those obtained from frozen and FFPE tissues. Their results showed that specific miRNAs can be similarly recovered from frozen, thawed, and FFPE tissue samples, indicating a high level of molecular stability. In fact, as also explained by Li et al. [15], miRNAs are stable molecules because of their short length (20–22 nt) and because they are bound to proteins of the RISC complex, which protect them from degradation. In this work, cells were processed as FFPE samples: suspended cells were aliquoted, pelleted, and either snap-frozen or FFPE to generate cell blocks. A limitation of this early study is that it did not analyze tissue collected directly from subjects but instead relied on cultured cells that were subsequently fixed in formalin. Following this initial investigation, several subsequent studies using paraffin-embedded tissue samples confirmed the stability of miRNAs.

Peiro-Chova et al. in 2013 [16] conducted the first study in which the stability of small RNA molecules (snoRNA and miRNAs) was analyzed in thawed tissue samples and then compared to that of frozen and FFPE tissue samples. The robustness and stability of miRNAs in FFPE tissue were tested. The researchers analyzed 14 cryopreserved tissue samples, 10 frozen samples that underwent a severe thawing process and their paired FFPE tissue samples from patients with breast cancer obtained during primary surgical resection. Researchers performed an electrophoresis of the three groups of samples (optimally frozen, thawed, and FFPE). The electrophoretic profiles of small RNAs from improperly frozen and thawed samples, as well as from FFPE tissues, were compared with those from properly stored fresh-frozen tissues. Specific miRNA molecules resulted similarly recovered from the three different tissue sample sources, supporting their high degree of stability. Well-preserved fresh-frozen tissues represent the optimal material for molecular analyses and high-throughput technologies; however, their availability in large quantities is often limited. Consequently, alternative tissue sources such as FFPE samples are increasingly used, as they preserve tissue morphology effectively and are widely available in pathology and histology archives worldwide, representing a valuable resource for biomedical research. Furthermore, the storage of frozen tissues is costly and labor-intensive and carries the risk of accidental events, such as uncontrolled thawing at room temperature, which may compromise sample integrity.

Furthermore, qRT-PCR analysis was performed to evaluate the recovery of three representative miRNAs (miR-21, miR-125b, and miR-191) in optimally frozen, thawed, and FFPE samples. Interestingly, the FFPE specimens exhibited lower Ct values than both frozen and thawed tissues, suggesting a higher apparent recovery of these miRNAs. This finding is likely attributable to differences in the RNA extraction protocols adopted for FFPE and frozen samples rather than to intrinsic differences in miRNA abundance. In FFPE tissues, incomplete reversal of formalin-induced RNA–protein cross-links, despite proteinase K digestion, may hinder the extraction of longer RNA species while having a much smaller impact on short RNA molecules such as miRNAs, thereby contributing to their relatively efficient recovery. Peiro-Chova et al. [16] demonstrated that specific miRNA molecules are similarly recovered in different tissue sample sources (frozen, thawed, and FFPE), which supports their high degree of stability.

Another study on miRNA stability in FFPE tissue samples focused on guanine (G) and cytosine (C) content. Kakimoto and colleagues [17] showed that miRNAs with a higher GC% were better preserved in FFPE samples. Additionally, miRNAs with a GC% of less than 40% were significantly degenerated in FFPE specimens (*p* = 1.4 × 10^−10^). They, previously, demonstrated that the read length of RNA is strongly related with its stability in FFPE samples, and miRNAs are more abundantly detected than longer RNAs after prolonged fixation [18]. Subsequently, they compared the stability of miRNAs in FFPE cardiac tissues using NGS. The mode read length in FFPE samples was 11 nucleotides, while that in the matched frozen samples was 22. Deep sequencing and qPCR analyses demonstrated that GC content affects the stability of miRNAs in FFPE samples. This study further showed that miRNA degradation occurs predominantly at the 3′ end rather than the 5′ end. Identified miRNA-degrading enzymes include both 3′-to-5′ and 5′-to-3′ exoribonucleases, while no endoribonucleases have been reported. Exoribonuclease activity may differ between the two ends, and the absence of a poly-A tail at the 3′ end may further promote degradation after tissue sampling. Nonetheless, the precise mechanisms governing miRNA turnover remain largely unknown.

Mucciaccia et al. [19], in 2015, compared the degradation of different types of tissues. The study aimed to assess whether small RNA fractions isolated from human tissues collected during autopsy at different post-mortem intervals (PMIs) are suitable for forensic molecular analyses. To this end, the authors examined FFPE tissue samples, including post-mortem and putrefied specimens, obtained from four autopsy cases with varying PMIs. RNA recovery, quality, and integrity were evaluated, together with the suitability of the extracted RNA for forensic applications, using reverse transcription quantitative polymerase chain reaction (RT-qPCR). In addition, their findings indicated that the progression of RNA degradation over time is influenced by the anatomical origin of the tissue. More recent studies have further demonstrated that post-mortem RNA stability varies among tissues [20,21], likely because of differences in the abundance and activity of endogenous ribonucleases and other degradative enzymes across organs. Evaluation of RNA integrity—assessed through RNA Integrity Number (RIN) values in total RNA isolated from fresh, unfixed mouse tissues collected at increasing intervals after euthanasia—revealed that cardiac and pulmonary tissues retained the highest RNA quality, even after extended post-mortem periods. Previous studies have shown that post-mortem nucleic acid degradation begins rapidly in abdominal organs, where chemical, thermal, and microbial processes are activated soon after death. Consequently, RNA integrity declines more quickly in these tissues than in organs such as the lung and brain, which are not exposed to resident bacterial flora. In contrast, miRNAs exhibit greater post-mortem stability because of their short length and their association with protein complexes that protect mature miRNAs from degradation, making them considerably more resistant than mRNAs. Furthermore, miRNA expression patterns are minimally affected by FFPE and remain highly comparable to those obtained from fresh or frozen specimens. Comparative analyses of total RNA isolated from different tissues have also demonstrated that RNA degradation follows tissue-specific patterns that correlate with the PMI. As degradation progresses, the relative proportion of small RNA species increases, reflecting the accumulation of short RNA fragments generated during the degradation process.

Boisen et al. [22] published a study that further confirmed the stability of miRNAs under different formalin fixation conditions. While formalin fixation caused a reduction in miRNA expression, the expression profiles of frozen and FFPE samples from the same tumor remained largely highly correlated. Four sections of fresh colon rectal cancer (CRC) and pancreatic (PC) tissues were considered to compare miRNA expression in different conditions. One section was frozen at −80 °C, the others were fixed in 10% of formalin-fixation solution for 2, 3 and 6 days respectively. After fixation, tissue sections were embedded in paraffin and then stored at room temperature. Overall, considerable variability between frozen and FFPE samples was observed, with frozen samples from different tumors often clustering together in hierarchical analyses. Global mean normalization had limited impact on improving clustering, suggesting that formalin fixation may induce non-uniform alterations in miRNA measurability. Although formalin fixation for 2–6 days reduced miRNA expression by 30–65%, expression profiles from frozen and matched FFPE samples from the same tumor generally remained highly correlated. Many previous studies have reported a good correlation between frozen and FFPE samples [23,24,25,26,27,28]. Building on early studies that demonstrated the stability of miRNA molecules even in complex biological matrices such as FFPE tissues, other studies [13,14] extended the research by examining the long-term persistence of miRNA stability over time.

Bovell [14] and colleagues were the first to demonstrate the stability of miRNAs in FFPE tissue samples stored for long periods (>20 years). In their investigation, the authors evaluated the expression of six miRNAs (miR-20a, miR-21, miR-106a, miR-181b, miR-203, and miR-324-5p) in 345 FFPE colorectal cancer (CRC) specimens (collected between 1982 and 2004) using TaqMan MicroRNA assays combined with quantitative RT-PCR. The analysis revealed that the expression of all six miRNAs remained comparable across samples regardless of storage duration, including those preserved for more than 28 years. Statistical evaluation showed no significant differences in miRNA expression levels between tissues with different storage times (all *p* > 0.05), indicating that long-term FFPE preservation did not adversely affect miRNA abundance. Moreover, regression analyses failed to identify any association between miRNA expression and the year of tissue collection, further supporting the long-term stability of miRNAs in archived FFPE colorectal cancer specimens.

Subsequently, Peskoe, in 2017 [13] collected 92 FFPE radical prostatectomy specimens stored for 12–20 years. Transcript stability was evaluated by applying general linear models to examine changes in expression over time, while the relationships among transcript abundance, specimen age, and RNA Integrity Number (RIN) were assessed using Spearman’s rank correlation analysis. The authors also highlighted that RNA preservation in FFPE tissues can be influenced by several pre-analytical and storage-related factors, including fixation duration, fixation procedures, and exposure to environmental conditions such as oxidation, elevated temperatures, and light, all of which may contribute to progressive RNA degradation [13,29,30]. An interesting observation from this study concerns the differential stability of individual miRNAs. For example, miR-221 and miR-141 were significantly more stable over time compared to miR-21 and RNU6B. This supports previous reports of differential stability between different miRNAs [26]. No correlation was observed between sample RIN and the age of FFPE blocks. The study reported a gradual, linear decline in miRNA signal in FFPE samples stored for 12–20 years, with some miRNAs exhibiting greater stability than others. Overall, the age of the FFPE block emerged as the most consistent factor influencing miRNA stability. These results suggested that it would be beneficial to consider sample block age, rather than RNA quality, in miRNA expression analyses from older FFPE samples.

All studies mentioned have demonstrated the stability of miRNAs in FFPE tissues, promoting further utilization of stored tissue samples in the comprehensive analyses of these short molecules.

## 4. miRNA Molecular Analysis in FFPE Heart Tissue Samples

For the reasons outlined above, miRNAs are considered excellent biomarkers for a wide range of diseases, from cancer to cardiovascular disorders. Due to their high stability, their biomarker potential is preserved even when complex matrices, such as FFPE tissues, are used. In the forensic field, the identification of novel biomarkers may assist in the evaluation of complex causes of death, including SCD. Fresh tissues or blood samples are often unavailable in forensic investigations; therefore, the analysis of miRNA profiles from FFPE tissues represents a valuable alternative.

This narrative review highlights the relevance of miRNAs as molecular biomarkers capable of improving analytical outcomes even when derived from complex biological matrices such as FFPE tissues. In particular, forensic studies frequently rely on paraffin-embedded tissue blocks for the investigation of SCD. This review summarizes the scientific literature published to date on miRNA analyses performed on FFPE cardiac tissues, employing a range of methodologies from conventional techniques, such as real-time PCR, to advanced approaches, including NGS.

Among the different studies included in the review, miRNAs were analyzed through different techniques. The amount of biological material collected is variable—the number of sections considered for extraction and even their thickness. Section thickness can range from 4 to 20 µm [17,18] and the number of sections used for miRNA extraction varies from one to two [17,18], up to eight slices of embedded tissue [31,32,33,34,35,36,37,38,39,40].

Before proceeding with the isolation and extraction of nucleic acids, it is necessary to remove as much paraffin as possible from the FFPE tissue block because it could interfere with the extraction yield. One of the most commonly used approaches for this purpose is xylene. This approach is still highly debated. It is intended to remove paraffin, which interferes with the recovery of biological material. Nonetheless, the use of xylene prior to nucleic acid extraction is often considered controversial and, in some cases, undesirable for several reasons. Exposure to organic solvents such as xylene may lead to partial degradation or the loss of nucleic acids, particularly small RNAs, thereby reducing overall yield. In addition, xylene-based protocols have been associated with increased variability in RNA yield and quality among samples, which may affect the reliability of downstream quantitative analyses such as qPCR or NGS. Furthermore, xylene is highly toxic, volatile, and flammable, requiring strict safety measures and specialized laboratory infrastructure, which limits its suitability for routine diagnostic or forensic workflows. Moreover, the inclusion of xylene can complicate protocol standardization, whereas alternative deparaffinization approaches tend to be simpler and more reproducible.

Importantly, recent studies have demonstrated that xylene-free deparaffinization methods—such as heat-based protocols or the use of less aggressive, proprietary reagents—can provide miRNA yields and quality comparable to, or in some cases superior to, those obtained with xylene, thereby questioning its necessity in modern extraction protocols [35,36,37,38,39,40,41].

The extraction kit most used in papers selected for this review is the miRNeasy FFPE kit (Qiagen, Hilden, Germany) [19,31,32,33], the High Pure miRNA Extraction Kit (Roche, Mannheim, Germany) [36] and the RecoverAll Total Nucleic Acid Isolation Kit (Applied Biosystems, Waltham, MA, USA) [17,18,37,38].

Extraction kits could have different performances and different yields, and there are no comparisons between kits.

The first research study investigating miRNAs in FFPE cardiac tissues was conducted by Boštjančič et al. [31] in 2012. They cut three to eight sections of 10 µm and the total RNA was isolated using the miRNeasy FFPE kit (Qiagen, Hilden, Germany). They added xylene for deparaffinization. The concentration of extracted RNA was measured by NanoDrop-1000 (Thermo Scientific, Waltham, MA, USA) and the integrity was analyzed on a Bioanalyzer 2100 (Agilent, Santa Clara, CA, USA).

They analyzed total RNA from three infarcted tissues and compared it with three corresponding remote myocardium samples. Five to ten μg of RNA from heart samples was used for miRNA microarray analysis. The miRNA expression profiling revealed 43 differentially expressed miRNAs in infarcted tissue compared with the corresponding remote myocardium. Twenty miRNAs showed expression levels similar to those observed in healthy human hearts, 19 exhibited greater differences in expression, and four were uniquely differentially expressed in the present study.

The results were validated using two different qPCR technologies: SYBR Green and a TaqMan-based approach. A subset of miRNAs identified as differentially expressed by microarray analysis was confirmed by qPCR. Validation was performed using the miScript system (Qiagen, Hilden, Germany) or TaqMan-based technology (Applied Biosystems, Waltham, MA, USA). For this purpose, two pooled RNA samples were generated from RNAlater-preserved and FFPE tissue samples. The authors demonstrated that expression analyses performed using TaqMan or SYBR Green yielded comparable results and that miRNA expression data obtained from RNAlater-preserved and FFPE tissues were consistent across both technologies. Given these comparable results, FFPE tissue presents clear advantages over samples preserved in RNAlater at −20 °C, including reduced costs and storage requirements. Additionally, FFPE blocks are suitable for both genetic analyses and histological examination.

The study from Courts et al. [32] is the first to assess whether miRNA expression can be reliably measured in FFPE tissues from SIDS patients and whether potentially pathogenic dysregulation of heart- and brain-specific miRNAs can be detected. Twenty-eight samples of heart tissue were collected from 14 German SIDS patients and 14 controls (children who died from causes other than SIDS). Fresh tissues were immediately frozen, transported on dry ice when necessary, and then stored at −80 °C until further processing. The FFPE tissue collective consisted of samples of brainstem tissue from 11 German SIDS patients and 10 age-matched German controls. For extraction from FFPE tissues, slices of 10 mm were cut from the tissue blocks. Total-RNA containing small-RNA was extracted from FFPE tissue slices using the miRNeasy FFPE Kit (Qiagen, Hilden, Germany). Total-RNA concentration was measured with the QubitTM fluorometer (Invitrogen, Waltham, MA, USA) using the Quant-iTTM RNA Assay Kit (Invitrogen, Waltham, MA, USA). Then, miRNAs were reversely transcribed to cDNA using the TaqMan1 MicroRNA Reverse Transcription Kit (Applied Biosystems, Waltham, MA, USA). The kit utilized a specific priming strategy in which miRNA specific stem loop primers bind to their mature miRNA target; thus, only mature miRNAs are transcribed to cDNA. MiRNA expression levels were quantified using qPCR on an ABI PRISM 7000 Sequence Detection System (Applied Biosystems, Waltham, MA, USA), employing TaqMan miRNA Assays (Applied Biosystems, Waltham, MA, USA). Two small nucleolar RNAs (snoRNAs), U18 and U47, were considered for subsequent normalization of non-biological variances in the data. Statistical analyses included assessment of data normality, comparison of mean expression levels using Student’s *t*-test or the Mann–Whitney U test, and Dunn–Bonferroni correction for multiple testing; *p* ≤ 0.05 was considered statistically significant. For each tissue they obtained different results.

Expression analysis revealed a significant upregulation of miR-1 in fresh cardiac tissue obtained from children with Sudden Infant Death Syndrome compared with control subjects, whereas no significant differences were observed in miR-133a expression between the two groups. In FFPE brainstem specimens, let-7b expression was also significantly increased in SIDS cases relative to controls, while miR-124a levels remained comparable between groups. Based on these findings, the authors proposed that tissue-specific alterations in miRNA expression, particularly the dysregulation of miR-1 in the heart and let-7b in the brainstem, may contribute to the pathophysiology of SIDS. The dysregulation of miR-1 observed in post-mortem SIDS cases is consistent with extensive evidence obtained in living patients and experimental models [42,43]. miR-1 is one of the best-characterized cardiac miRNAs and plays a key role in cardiac electrical conduction by regulating ion-channel expression, calcium handling, and gap-junction proteins, particularly connexin-43. Altered miR-1 expression has been associated with increased arrhythmogenic susceptibility in several cardiovascular disorders, supporting the biological plausibility of its involvement in SCD rather than representing a post-mortem phenomenon alone. Similarly, miR-133a contributes to cardiac electrical stability and myocardial remodeling and has also been implicated in inherited and acquired arrhythmogenic disorders.

Mucciaccia et al. [19] analyzed amplification performance of FFPE-extracted RNA, showing that it is specific for different organs from the same individual. This pilot study showed that RNA profiles obtained from different organs (brain, liver, lung, kidney and myocardium) and at different post-mortem intervals provide valuable insights and considerations, highlighting their potential applications in forensic science.

Their work demonstrated that, despite the marked and heterogeneous degree of RNA degradation, total RNA could still be successfully recovered from FFPE samples of all the post-mortem organs examined, allowing subsequent molecular analyses of small RNA targets.

RNA was isolated using the miRNeasy FFPE Kit (Qiagen, Hilden, Germany). RNA yield and purity were assessed by measuring absorbance at 260 nm with a NanoDrop ND 1000 spectrophotometer (Thermo Scientific, Waltham, MA, USA), while RNA quality and integrity were evaluated by on-chip microcapillary electrophoresis using an Agilent 2100 Bioanalyzer with the RNA 6000 Nano Kit (Agilent Technologies, Santa Clara, CA, USA). RT-qPCR analysis was performed using two different commercially available gene expression kits. In particular, reverse transcription of total RNA was performed using the High Capacity RNA-to-cDNA kit (Applied Biosystem, Waltham, MA, USA) or the miScript II RT kit (Qiagen, Hilden, Germany), depending on the type of RNA target to be amplified, following the manufacturer’s recommendations. After an extensive evaluation of RNA integrity across different tissue types, the research group focused on miRNAs, as they are specifically preserved even after long post-mortem intervals. In particular, miR-21 was detected in all post-mortem samples analyzed—including putrefied organs—across different post-mortem intervals, suggesting that miRNAs represent one of the few molecular targets that can be further investigated for forensic purposes. The amplification of specific miRNAs from FFPE tissues could therefore facilitate retrospective molecular analyses aimed at determining the timing of injuries and myocardial infarction, as well as supporting the differential diagnosis of asphyxial death. This approach could be applied to archived fixed tissues in forensic settings and may provide additional evidence in cases where fresh or frozen material is no longer available.

Being the central theme of the review, we want to focus on the results on myocardial tissue. miR-21 was consistently expressed in all the post-mortem conditions analyzed, including in cases showing signs of putrefaction, where it was in fact the tissue exhibiting the highest levels of miR-21 expression. These findings suggest that cardiac tissue may represent one of the most suitable targets for miRNA analysis, even at extended post-mortem intervals and following formalin fixation. These results showed that the identification of specific miRNAs in heart tissue samples may help elucidate the presence of myocardial infarction or other cardiac-related diseases.

In the study carried out by Kakimoto et al. in 2015 [18], archival tissue samples were utilized to establish a method to evaluate the miRNA profiles of autoptic tissues following exposure to rough conditions in order to elucidate potential biomarkers of acute myocardial infarction (AMI). They suggested that cardiac tissue is particularly suitable for post-mortem quantitative analyses, as it undergoes less post-mortem degradation than many other organs. Nineteen samples of frozen cardiac tissue and 36 samples of FFPE were collected. Over-fixed samples, which were those formalin-fixed for more than three months, were excluded from the study, as the longer fixation periods appeared to increase the degradation of candidate small RNA.

Total RNA was isolated with a RecoverAll Total Nucleic Acid Isolation Kit (Applied Biosystems) adding a step of deparaffininization at 50 °C for 5 min and modifying some steps as described in the manuscript [18]. RNA concentration and purity were evaluated with a spectrophotometer (BioSpec-nano, Shimadzu, Kyoto, Japan). RNA integrity was assessed using microcapillary electrophoresis on a 2100 Bioanalyzer with a Small RNA kit (Agilent Technology, Santa Clara, CA, USA). Four candidate biomarkers were selected for the study: miR-1, miR-133a, miR-208b, miR-499a-5p. As control candidates, three miRNAs (miR-191, miR-93, miR-26b) and three other small RNAs were selected. These miRNAs are specific to cardiac muscle and relevant for AMI diagnosis. cDNA was synthesized using a TaqMan MicroRNA Reverse Transcription kit (Applied Biosystems, Waltham, MA, USA) according to the manufacturer’s instructions. Subsequently, mature miRNAs were quantified using a StepOnePlus real-time PCR system (Applied Biosystems, Waltham, MA, USA). Data processing was performed using StepOnes software version 2.3 (Applied Biosystems, Waltham, MA, USA). Comparisons between the AMI cases and controls were performed with the Mann–Whitney U-test (*p*-values < 0.05). miR-133a was excluded from further analysis due to its grossly outlying amplification efficiency (more than 10%). miR-208b was the most stable miRNA over increasing fixation times, while initially more abundant miRNAs—miR-26b, miR-1, and miR-499a—gradually decreased below its levels after 11, 36, and 39 months of fixation, respectively. miR-191 and miR-26b demonstrated to be the best endogenous control genes to quantify the expression of post-mortem miRNA biomarkers in deceased cardiac infarction patients, since they were less influenced by the PMI or fixation time of the sample compared to the other control gene candidates. The method described by Kakimoto et al. suggested that miRNA quantification of FFPE tissues is practically useful at post-mortem examination, and can be a helpful diagnostic tool for critical cardiac injury.

In 2016, Kakimoto et al. [17] performed a second study analyzing miRNA deep sequencing. The small RNA libraries were created using the Ion Total RNA-seq kit v2 (Life Technologies, Carlsbad, CA, USA), according to the manufacturer’s instructions. Libraries were, then, sequenced on an Ion 318 chip using the Ion Torrent PGM system (Life Technologies, Carlsbad, CA, USA). Sequencing data was analysed by Torrent Browser. From the three frozen specimens, a total of 6,462,853 sequencing reads were obtained, of which 5,979,759 (92.1%) successfully aligned to the human reference genome (hg19). Among these aligned reads, 2,080,072 (32.0%) corresponded to annotated miRNAs listed in miRBase v21. Conversely, sequencing of the three FFPE samples produced 11,388,982 reads in total, with 9,905,331 reads (87.0%) mapping to the human genome; however, only 1,070,701 reads (9.4%) were assigned to known miRNAs. Notably, the FFPE sample that underwent the longest fixation time exhibited the poorest miRNA mapping efficiency (5.6%). Differences in miRNA expression were observed between the paired samples. In frozen tissues, miR-1-3p was the predominant miRNA, accounting for approximately 20–30% of the total reads. By comparison, FFPE samples were characterized by a different dominant miRNA, miR-133a-3p, which comprised up to 14% of the overall miRNA population, whereas miR-1-3p contributed less than 10% of total miRNA reads in FFPE specimens.

To validate the results of miRNA deep sequencing, the researchers performed qPCR analysis on seven selected target miRNAs with a GC% of 27–64% detected in the cardiac tissue in abundance.

Although miRNAs are more stable than mRNAs, the data demonstrated some limitations of FFPE sample utilization for miRNA deep sequencing. Because miRNAs degrade at different rates, the relative expression of each miRNA in FFPE tissue may not match that in the corresponding frozen tissue. Reliable quantification should be supported by additional analyses, such as qPCR validation, and employing multiple quantitative approaches may enhance the future use of archival FFPE samples.

Subsequently, Di Francesco et al. [33] applied NGS technology in FFPE endomyocardial biopsies (EMBs) to explore the miRNA expression profile of heart transplanted patients. The results were validated using a real-time PCR.

The cited study is not intended for forensic purposes; however, it highlighted the relevance of using formalin-fixed, paraffin-embedded cardiac tissue in other clinical contexts without requiring repeated endomyocardial biopsies exclusively for molecular analyses. It provides valuable methodological evidence supporting the use of FFPE specimens for retrospective molecular analyses, despite its limited direct applicability to SCD investigations.

Li et al. [35] showed the diagnostic value of cardiac miR-126-5p, miR-134-5p and miR-499a-5p in CAD-SCD cases. The study included 30 cases of coronary artery disease-related sudden cardiac death (CAD-SCD), comprising 18 individuals with a history of recurrent asymptomatic myocardial ischemia (CAD-activated SCD) and 12 cases lacking evident pathological signs of insufficient myocardial perfusion (CAD-silent SCD). An additional 30 victims of traumatic death were recruited as the control group. Total RNA was isolated from FFPE tissue specimens using the miRNeasy FFPE Kit (Qiagen, Hilden, Germany) after sectioning each tissue block into two or three slices, each 10 μm thick.

The expressions of cardiac miR-126-5p, miR-134-5p, and miR-499a-5p were analyzed by RT-qPCR, using miRCURY LNA RT Kit (Qiagen, Hilden, Germany) and miRCURY LNA SYBR Green PCR Kits (Qiagen, Hilden, Germany) according to the instructions of the manufacturer. U6 snRNAs were used as the endogenous control for miRNA quantification. Results showed a downregulation of miR-126 5p in CAD-SCD victims by ~3.1-fold an a downregulation of miR-499a-5p by ~1.9-fold. There was no statistically significant trend in miR-134-5p between the two groups. The authors concluded that the combination of miR-126-5p and miR-499a-5p is a good indicator for assessing CAD-SCD, demonstrating strong diagnostic performance. The differential expression of miR-126-5p and miR-499a-5p identified in CAD-SCD is also supported by clinical evidence. MiR-126 is predominantly expressed in endothelial cells and contributes to vascular homeostasis and endothelial repair, whereas reduced circulating levels have been associated with coronary artery disease and endothelial dysfunction. Conversely, miR-499 is highly enriched in cardiomyocytes and is released following myocardial injury, making it one of the most extensively investigated biomarkers of acute myocardial infarction. Therefore, the altered expression observed in post-mortem FFPE cardiac tissue is coherent with the biological functions previously demonstrated in clinical cardiovascular studies [44,45].

Koussa et al. [34] also applied miRNA analysis in a clinical setting. The authors performed a targeted investigation to identify differentially expressed miRNAs in post-mortem lung and cardiac tissues obtained from infants with bronchopulmonary dysplasia (BPD). Total RNA was isolated from FFPE tissue sections following deparaffinization, using the miRNeasy FFPE Kit (Qiagen, Hilden, Germany). RNA concentration was subsequently determined with a NanoDrop ND-1000 spectrophotometer (Thermo Fisher Scientific, Waltham, MA, USA). In this study, a different technique was employed—one that is widely used for the analysis of miRNA expression—namely, miRNA microarray analysis, using Agilent G3 Human v21 8X60K miRNA arrays. This study identified miRNAs that are similarly dysregulated in archived post-mortem lung and heart samples in subjects with histologic BPD. The results showed upregulation in miR-378b, miR-184, miR-3667-5p, miR-3976, miR-4646-5p, and miR-7846-3p across both tissue types. The interpretation and prediction of targets for the miRNAs of interest rely on bioinformatic prediction tools, such as DAVID and mirDIP, as well as pathway prediction resources, including KEGG pathway analysis.

More recently, studies on SCD using FFPE heart tissue have gained increasing attention, and, in particular, two notable studies have been published. The first, by Mildeberger et al. [35], aimed to identify novel reliable biomarkers to differentiate among cardiac death cases. In this study, the potential of various miRNAs as biomarkers was evaluated in both tissue and blood samples from cases of cardiac death. Slices of FFPE were used for the RNA extraction, which was performed with the High Pure miRNA Extraction Kit (Roche, Basel, Switzerland). A NanoDrop Spectrophotometer (Thermo Scientific, Waltham, MA, USA) was used for measuring the concentration. Quantification of the miRNA was performed using the TaqMan MicroRNA Assay (Applied Biosystems, Waltham, MA, USA) in a two-step RT-PCR. The results showed that miR-1, miR-133a and miR-26a were upregulated in FFPE tissues of the SCD cases compared to the myocardial infarction and control cases. The upregulation of miR-26a is also statistically significant. All three miRNAs showed great potential to discriminate between the causes of SCD.

The last study on post-mortem analyses of myocardial miRNA expression in FFPE heart tissue is the one conducted by Lehto et al. [37]. They published the first study investigating miRNA expression using FFPE cardiac tissue from patients with sepsis.

Total RNA was extracted from FFPE cardiac tissue using the RecoverAll™ Total Nucleic Acid Isolation Kit for FFPE (Invitrogen, Waltham, MA, USA). MicroRNA expression profiling was subsequently performed by NGS. Sequencing libraries were prepared with the RealSeq Single Index Kit (RealSeq Biosciences, Santa Cruz, CA, USA) and analyzed on the Illumina NovaSeq SP100 (Illumina, Inc., San Diego, CA, USA) platform using single-end sequencing. Bioinformatic processing of the sequencing data was carried out with the miND^®^ and meND v1.2.8 analysis pipelines. Differential miRNA expression was assessed using edgeR v3.32, while functional and pathway analyses were conducted with Ingenuity Pathway Analysis (IPA; Qiagen), integrating target predictions from the TargetScan Human, Ingenuity Expert Findings, miRecords, and TarBase databases. MiRNA sequencing identified a total of 1753 miRNAs, of which 267 showed high expression levels in the samples and were therefore selected for differential expression analysis. This analysis revealed 32 miRNAs that were differentially expressed in myocardial tissues from sepsis cases compared with control samples. Among the eight miRNAs with a log_2_FC > 1, miR-12136 showed the largest increase in mean counts in septic heart tissue, miR-146b-5p had the highest fold change, and miR-451a had the largest difference in mean counts and highest fold decrease. Multiple regulatory miRNAs showed significant up- or down-regulation in the myocardial tissue of septic patients compared with non-septic individuals. Notably, all the miRNAs discussed have been previously associated with inflammatory processes or cardiovascular pathologies.

## 5. The Challenge of Endogenous Reference Gene Validation in FFPE Tissue

The qualitative and quantitative analysis of miRNA expression within FFPE cardiac samples represents a pivotal milestone for molecular autopsies.

A major methodological vulnerability identified across the included literature concerns the heterogeneity of reference genes utilized for data normalization. The reviewed studies rely on highly diverse internal controls, ranging from small non-coding RNAs like U6 snRNA, U18, U47, and RNU6B, to specific mature miRNAs such as miR-191 and miR-26b. In a post-mortem matrix, the selection of an unvalidated reference gene carries the distinct risk of masking true pathophysiological variations or generating artifactual results. Although miRNAs are remarkably stable, as discussed above, they are not completely resistant to degradation or chemical modification. Such alterations may result from prolonged formalin fixation or from post-mortem degradation in tissues collected after death and subsequently embedded in paraffin. Furthermore, endogenous reference small RNAs are also susceptible to degradation, often exhibiting degradation kinetics that differ from those of the target miRNAs. As a result, the expression levels of commonly used endogenous reference RNAs may vary unpredictably with the PMI, thereby introducing normalization bias and potentially affecting the reliability of miRNA expression analyses.

This issue is further exacerbated by the current lack of universally validated normalization strategies for miRNA quantification. Rather than relying on conventionally used endogenous controls, recent evidence [46] highlights the need to systematically validate candidate reference genes within each specific experimental setting, as their expression stability is highly dependent on tissue type, biological condition, and pre-analytical variables. The absence of standardized and context-specific normalization approaches remains a major source of variability, limiting the reproducibility and comparability of miRNA expression studies.

A mathematically sound reference strategy requires demonstrating that the internal control maintains structural stability and statistical independence from the PMI, exhibiting a low coefficient of variation across both the putrefied and control samples. For instance, Kakimoto et al. [18] critically addressed this gap by evaluating six candidate controls, concluding that miR-191 and miR-26b represent the most reliable endogenous references for autoptic cardiac investigations, as they remained entirely unconditioned by PMI progression or formalin fixation artifacts compared to traditional small nuclear RNAs. Future forensic profiling must systematically incorporate multiple-reference validation algorithms to prevent pre-analytical degradation from confounding diagnostic yield.

## 6. Pre-Analytical Factors Affecting miRNA Stability in FFPE Cardiac Tissue: Current Limitations

Despite the undeniable structural resilience of miRNAs within archival FFPE cardiac matrices, advancing this epigenetic tool into routine forensic practice and judicial cross-examinations requires overcoming several critical limitations and translational bottlenecks. The foremost challenge resides in the absolute lack of methodological standardization. Across the reviewed literature, there is high heterogeneity in tissue-to-formalin ratios, fixation durations (ranging from 24 h to several weeks), and paraffin block age. Formalin fixation induces progressive methylene bridges and covalent cross-linking between proteins and nucleic acids, causing highly variable RNA extraction yields and unpredictable chemical modifications. Furthermore, the extraction protocols themselves differ significantly in their capacity to recover the small RNA fraction without losing low-molecular-weight transcripts, making inter-study quantitative comparisons mathematically fragile. This remarkable heterogeneity of RNA extraction protocols, library preparation methods and normalization strategies is an important limitation. Because no study directly compared different extraction procedures using identical FFPE cardiac specimens, it remains impossible to determine which methodology provides the highest RNA yield, analytical sensitivity, or reproducibility.

In addition to analytical challenges, pre-analytical variables and the quality of the starting specimen must also be carefully considered. Factors such as tissue collection, handling, fixation protocol, fixation duration, storage conditions, and PMI can substantially influence the accuracy and reliability of miRNA analyses.

A fundamental methodological limitation in the current literature is the direct, uncritical extrapolation of miRNA findings from clinical settings to forensic post-mortem investigations. Clinical FFPE specimens—typically derived from endomyocardial biopsies or scheduled surgical resections—benefit from immediate stabilization, controlled ambient temperatures, and standardized processing protocols. Conversely, forensic autoptic tissues are subject to an entirely divergent set of pre-analytical degradation kinetics that systematically alter miRNA stability and expression signatures [47,48]. Crucially, the PMI introduces profound tissue autolysis, where hypoxia, warm ischemia, and microbial activity trigger the uncontrolled release of cellular hydrolytic enzymes [18,49]. Although miRNAs are partially shielded by their small size and Argonaut-protein associations, advanced autolysis progressively compromises their structural integrity, leading to artificial downregulation. This degradation pathway is further aggravated by the unpredictable duration for which a forensic corpse spends before body refrigeration, where the complete lack of cold-chain integrity accelerates environmental decay and introduces exogenous RNases before formal sampling can occur [49].

Furthermore, significant divergences emerge during the subsequent tissue processing stages. While clinical pathology workflows strictly regulate fixation time, forensic autopsies frequently suffer from prolonged, unmonitored fixation intervals due to weekend delays or legal mandates. Extended exposure to formalin causes extreme, irreversible methylene bridge formations, heavily restricting RNA extraction yields and modifying miRNA bases in a way that inhibits reverse transcription. Finally, whereas clinical biopsies target specific, uniform myocardial zones, forensic sampling must navigate massive tissue heterogeneity, including necrosis, putrefactive gas formation, and varying degrees of localized autolysis across different cardiac chambers, creating structural confounding factors entirely absent in clinical cohorts.

The evidence compiled in the reviewed studies unequivocally demonstrates that once a tissue specimen has been adequately fixed and embedded, its miRNA cargo remains remarkably stable for prolonged periods, with several studies reporting successful miRNA quantification even after decades of FFPE storage; however, this exceptional ex vivo stability should not be interpreted as evidence that miRNAs are intrinsically resistant to degradation under all conditions. Rather, it reflects the preservation achieved after tissue fixation and embedding, whereas the interval preceding fixation remains a critical phase during which RNA integrity may progressively decline.

This distinction is particularly relevant in forensic pathology, where PMI represents a major pre-analytical variable [50]. During the post-mortem period, cardiac tissue is exposed to hypoxia, ischemia, autolysis, and microbial activity, all of which contribute to progressive nucleic acid degradation. Consequently, the miRNA profile recovered from an autoptic FFPE specimen should be interpreted as the result of multiple biological and technical factors, including the individual’s underlying pathology, the duration of the post-mortem interval, and tissue processing procedures. These variables may introduce transcript-specific degradation patterns and influence miRNA abundance independently of disease-related molecular changes. Therefore, a better understanding of post-mortem RNA degradation kinetics and the development of models capable of distinguishing post-mortem effects from true biological variation remain essential prerequisites for the reliable implementation of miRNA-based biomarkers in forensic investigations.

## 7. Conclusions

SCD may result from a wide spectrum of underlying conditions, including structural cardiac diseases, inherited channelopathies, and cardiomyopathies, and its diagnosis is challenging for coroners. Post-mortem genetic testing plays a pivotal role in the investigation of SCD, particularly in cases of suspected inherited channelopathies and cardiomyopathies. Identifying pathogenic variants may not only contribute to establishing the cause of death but also enable cascade screening of at-risk relatives. Peripheral blood collected during autopsy represents the gold-standard biological specimen for post-mortem genetic analysis (molecular autopsy), as it provides high-quality genomic DNA suitable for sequencing; fresh-frozen tissue represents the preferred alternative when blood is unavailable. Unfortunately, a blood sample or fresh tissue is not always available for retrospective molecular investigations. In Italy, there are two different types of autopsy: one called a judicial autopsy and the other known as a diagnostic post-mortem examination. In general, it is more difficult to obtain consent for a diagnostic post-mortem examination than for a judicial autopsy, since the judicial autopsy does not require the consent of the family, as it is ordered by the judicial authority for legal and investigative purposes. For this reason, the issue of consent does not arise in practice. By contrast, a diagnostic post-mortem examination usually requires the consent of the deceased’s relatives. This is where most of the difficulties emerge. Family members may refuse consent due to emotional distress, cultural or religious beliefs, or a limited understanding of the clinical value of the procedure. In both the judicial autopsy and, especially, in the diagnostic post-mortem examination, genetic analysis is not mandatory, although it is recommended. This implies that, in some cases, physicians do not proceed with the collection of blood or fresh-frozen tissues for genetic testing, as these samples require storage at controlled temperatures (−20 °C or −80 °C), thereby increasing costs and demanding adequate freezer capacity within laboratory facilities.

Far more frequently, tissues are collected during autopsy and subsequently formalin-fixed for histological examination. This matrix does not require costly storage conditions, since FFPE tissues can be preserved at room temperature for extended periods. Although FFPE would be considered an ideal biological material for genetic analysis, albeit for the reasons just mentioned, formalin-fixation induces DNA-protein cross-linking, fragmentation and DNA damage which may lead to sequence alterations and spurious variant calls. The major issue is the occurrence of false positives caused by artifacts introduced during formalin fixation.

For all the reasons listed above, other genetic biomarkers that are more conservative, robust and stable may be considered. In the forensic setting, differential miRNA expression has been investigated to support the differential diagnosis of sudden cardiac death, identify myocardial ischemia or infarction, distinguish specific cardiac pathologies, and complement molecular autopsy when conventional genetic material is unavailable or severely degraded. In fact, miRNAs are highly stable, as both their expression levels and sequences remain unchanged even if the biological material is under adverse temperature or pH conditions. Many studies have focused on miRNAs and their role in cardiovascular diseases, owing to their differential expression patterns and intrinsic properties. In this review, we have summarized studies investigating miRNAs as stable biomarkers in FFPE heart tissue, with particular attention to their involvement in SCD and related cardiovascular conditions.

This work highlights that miRNAs have strong potential as valuable biomarkers due to their stability even in complex biological matrices such as FFPE tissues. This feature is fundamental for post-mortem studies, especially when FFPE is the only biological matrix available, remaining stable in long-term archived FFPE tissue blocks.

Although the available evidence supports the feasibility and analytical robustness of miRNA analysis in FFPE cardiac tissue, it should be acknowledged that the current literature is still heterogeneous with respect to specimen origin. Moreover, most of the published studies are exploratory in nature and were not specifically designed as diagnostic accuracy studies, limiting the strength of the available evidence regarding their clinical or forensic applicability. These investigations cannot be directly translated to molecular autopsy, where prolonged post-mortem intervals, autolysis, tissue decomposition, and variable fixation conditions represent additional pre-analytical challenges. Consequently, further studies specifically designed on well-characterized forensic cohorts are needed to validate the diagnostic applicability of these biomarkers under real post-mortem conditions.

## Data Availability

No new data were created or analyzed in this study.

## Supplementary Materials

The following supporting information can be downloaded at: https://www.mdpi.com/article/10.3390/genes17070819/s1, Table S1: Synoptic Overview of Included Studies on Myocardial microRNA Expression in FFPE and Post-Mortem Tissue.

## Abbreviations

SCD	Sudden Cardiac Death
FFPE	Formalin-Fixed Paraffin-Embedded
miRNA	microRNA
DNA	Deoxyribonucleic Acid
RNA	Ribonucleic Acid
SUD	Sudden Unexplained Death
NGS	Next-Generation Sequencing
RT-qPCR	Reverse Transcription Quantitative Polymerase Chain Reaction
PCR	Polymerase Chain Reaction
qPCR	Quantitative Polymerase Chain Reaction
PMI	Post-Mortem Interval
SIDS	Sudden Infant Death Syndrome
AMI	Acute Myocardial Infarction
CAD	Coronary Artery Disease
CAD-SCD	Coronary Artery Disease-Related Sudden Cardiac Death
EMB	Endomyocardial Biopsy
BPD	Bronchopulmonary Dysplasia
RISC	RNA-Induced Silencing Complex
IPA	Ingenuity Pathway Analysis
DAVID	Database for Annotation, Visualization and Integrated Discovery
KEGG	Kyoto Encyclopedia of Genes and Genomes
miRBase	MicroRNA Database
ROC	Receiver Operating Characteristic
AUC	Area Under the Curve
CI	Confidence Interval
FC	Fold Change
snRNA	Small Nuclear RNA
