# Supplementary material for: From Old to Bold: Advancing microRNA Studies in Sudden Cardiac Death Through Molecular Analysis of FFPE Heart Tissue
Source: Genes (Basel). 2026 Jul 17;17(7):819. doi: 10.3390/genes17070819 (PMC13409910; doi:10.3390/genes17070819)
Supplement: Supplementary file 1 [file genes-17-00819-s001.zip › genes-4376448-supplementary.pdf]

Table S1. Synoptic Overview of Included Studies on Myocardial microRNA Expression in FFPE and Post-Mortem Tissue

Author & Year	Setting & Cohort	Methodology & Normalizer	Candidate miRNAs	Statistical Significance	Key Findings & Forensic Relevance
**Boštjančič et al., 2012**	Clinical/Autoptic study<br>6 cases<br>3 AMI vs. 3 Remote myocardium	Microarray and qPCR (TaqMan / SYBR)<br>Normalizer: RNU6B/U6, snoRNAs	43 differentially expressed miRNAs (10 upregulated, 33 downregulated); 9 miRNAs validated (miR-21, miR-122, miR-126, miR-1, miR-133, miR-125a, miR-125b, miR-98)	Student's *t*-test; significance threshold $p < 0.01$; data log_2-transformed and normalized using cyclic LOWESS. No effect size or 95% CI reported.	Demonstrated high concordance of miRNA expression profiles between FFPE and RNAlater-preserved specimens.
**Courts et al., 2013**	Forensic Case-Control study<br>14 SIDS vs. 14 Controls (Heart)<br>11 SIDS vs. 10 Controls (Brainstem)	qPCR (TaqMan)<br>Normalizers: U18 and U47	Cardiac miR-1: Significantly upregulated in SIDS vs. controls	Differential expression assessed by qPCR; significant differences reported ($p < 0.05$). Fold change, effect size and 95% confidence intervals were not reported.	Confirmed tissue-specific dysregulation of cardiac miR-1, highlighting its potential role in SIDS pathogenesis.
**Muciaccia et al., 2015**	Forensic study (Autoptic/Putrefied)<br>4 Individuals at different PMIs	RT-qPCR (TaqMan / miScript)<br>Normalizers: RNU6-2 (U6)	Not applicable; no differential expression analysis performed.	Not applicable	Myocardial tissue exhibited the highest levels of miR-21 expression even under advanced decomposition, validating it as a robust post-mortem marker.
**Kakimoto et al., 2015**	Forensic case/control study (AMI)<br>19 Frozen vs. 36 FFPE autoptics	RT-qPCR (TaqMan)<br>Normalizers: miR-191 and miR-26b	AMI biomarkers: miR-1, miR-208b, miR-499a.	miR-499a: 2.1-fold decrease, $p = 0.019$ (significant).<br>miR-1: 1.4-fold decrease, $p = 0.257$ (NS).<br>miR-208b: 1.2-fold	Identified miR-191 and miR-26b as the most stable endogenous controls unaffected by PMI or prolonged fixation.

				increase, $p = 0.527$ (NS).<br>No effect sizes or 95% confidence intervals reported.	
**Kakimoto et al., 2016**	Methodological Forensic study<br>10 Frozen vs. 10 Paired FFPE	NGS (Ion Torrent)<br>Validation: TaqMan RT-qPCR<br>Sequencing data normalized as reads per million mapped (RPM). For qPCR, miR-99b-5p (GC content 64%) was used as the internal control.	240 miRNAs were analyzed (≥10 RPM).<br>qPCR validation was performed on 7 cardiac miRNAs: miR-99b-5p, miR-133a-3p, miR-133b, miR-22-3p, let-7e-5p, miR-21-5p, and miR-1-3p.	miRNA expression profiles showed strong correlation between frozen and FFPE tissues (Pearson's $r = 0.88–0.92$).<br>GC content significantly affected miRNA stability (Dunnett's test, $p < 0.0001$).<br>miRNAs with GC <40% were significantly more degraded than GC-rich miRNAs (Mann–Whitney U test, $p = 1.4 \times 10^{-10}$).<br>No effect sizes or 95% confidence intervals were reported.	Revealed that miRNA degradation in FFPE is dependent on GC% content.
**Di Francesco et al., 2018**	Clinical setting (FFPE EMBs)<br>33 Heart transplanted patients; Discovery cohort: 20 FFPE EMBs; validation cohort: 13 FFPE EMBs.	NGS<br>Validation: RT-qPCR<br>Differential expression analysis performed using edgeR after library normalization.	2,275 mature miRNAs profiled by NGS. Differential expression identified 46 miRNAs (ACR vs MR), 21 miRNAs (ACR vs pAMR) and 2 miRNAs (MR vs pAMR). Twelve candidate miRNAs were selected for	RT-qPCR validation by ANOVA ($p < 0.05$; $^{**}p < 0.001$). Logistic regression models achieved 90% discrimination (MR vs others), 100% discrimination (MR vs ACR), 80%	Highlighted the diagnostic utility of FFPE endomyocardial biopsies to avoid repetitive invasive clinical sampling.

			validation, including miR-208a-5p, miR-126-5p, miR-135a-5p, miR-27b-3p, miR-29b-3p, miR-199a-3p, miR-302, and miR-144-3p.	discrimination (MR vs pAMR); 76.7% (ACR vs others), 80% (ACR vs pAMR); 90% (pAMR vs others), 100% (pAMR vs ACR), and 70% (pAMR vs MR). No effect sizes or 95% confidence intervals were reported.	
**Li et al., 2022**	Forensic case/control study (CAD-SCD) 18 CAD-activated SCD vs. 12 CAD-silent SCD vs. 30 Controls	RT-qPCR (SYBR Green) Normalizer: U6 snRNA	Three miRNAs (miR-126-5p, miR-134-5p, and miR-499a-5p)	CAD-SCD vs control: miR-126-5p: Downregulated by ~3.1-fold in CAD-SCD ($p < 0.05$); AUC = 0.76 (95% CI 0.64–0.88, $p < 0.001$); miR-499a-5p: Downregulated by ~1.9-fold ($p < 0.05$); AUC = 0.82 (95% CI 0.72–0.93, $p < 0.001$); 134-5p: AUC = 0.61, $p > 0.05$. No significant change<br><br>CAD-activated vs CAD-silent SCD: miR-126-5p: AUC = 0.74 (95% CI 0.56–0.92); miR-499a-5p: AUC = 0.75 (95% CI 0.57–0.94); combined miR-126-5p +	Demonstrated strong combined diagnostic performance of miR-126-5p and miR-499a-5p as indicators of coronary artery disease-induced SCD.

				miR-499a-5p: AUC = 0.82 (95% CI 0.66–0.99), sensitivity 91.7%, specificity 77.8%.	
**Koussa et al., 2023**	Clinical / Autoptic study<br>8 Infants with BPD vs 12 controls	miRNA Microarray (Agilent G3)<br>Bioinformatic prediction (DAVID, mirDIP, KEGG)<br>No endogenous normalizer	Significant upregulation of 6 miRNAs: miR-378b, miR-184, miR-3667-5p, miR-3976, miR-4646-5p, and miR-7846-3p	Differential expression was considered significant after multiple-testing correction (FDR < 0.05).<br>Pathway enrichment analysis identified the Hippo signaling pathway as the most significantly enriched ($P = 2.0 \times 10^{-6}$; $FDR = 6.1 \times 10^{-4}$).	Identified cross-tissue (lung and heart) miRNA dysregulation associated with histologic bronchopulmonary dysplasia.
**Mildeberger et al., 2023**	Forensic case/control study 41 AMI-related SCD vs 27 controls.	Two-step RT-qPCR (TaqMan)<br>Normalizer: miR-191	3 miRNAs were analyzed: miR-1, miR-133a, and miR-26a.<br>miR-1 and miR-133a were significantly upregulated in AMI-SCD compared with controls, whereas miR-26a was significantly downregulated.	Statistical Analysis: Shapiro–Wilk, Levene, Student's *t*, Welch, Mann–Whitney U; $p < 0.05$.<br>Diagnostic Performance:<br>miR-133a: AUC = 0.99 (whole blood, AMI vs controls);<br>miR-26a: AUC = 0.69 (FFPE tissue, AMI vs SUD);<br>miR-1: AUC > 0.60.	Established the discriminative power of cardiac-specific miRNAs to differentiate between SCD and AMI post-mortem.

**Lehto et al., 2024**	Clinical / Autoptic 12 sepsis-related deaths and 12 age- and sex-matched non-septic controls	NGS (Illumina NovaSeq) Pipeline: edgeR and IPA<br>Normalization based on reads per million (RPM)	28 differentially expressed miRNAs.<br>Upregulated (18): miR-10400-5p, miR-4488, miR-3196, miR-4508, miR-146b-5p, miR-3960, miR-320d, miR-320c, miR-4787-5p, miR-21-3p, miR-12136, miR-155-5p, miR-320b, miR-4449, let-7c-5p, miR-652-3p, miR-98-5p, miR-28-3p. Downregulated (10): miR-140-3p, miR-181a-3p, miR-423-3p, miR-93-5p, miR-181c-3p, miR-127-3p, miR-199b-5p, miR-486-5p, miR-199a-5p, miR-654-3p, plus miR-136-3p, miR-363-3p, miR-144-5p and miR-451a showing the greatest decreases.	A conservative ≥2-fold change threshold was adopted for biological relevance because of the limited sample size. Significant miRNAs included: miR-10400-5p (FDR=0.0014; log_2FC=3.5), miR-4488 (0.0024; 3.1), miR-3196 (0.0034; 2.3), miR-146b-5p (0.0042; 1.9), miR-155-5p (0.029; 0.98), miR-21-3p (0.035; 1.1), miR-451a (0.0014; −1.4), miR-144-5p (0.0016; −1.2), miR-363-3p (0.0014; −0.83), miR-486-5p (0.0042; −0.64), miR-199a-5p (0.0042; −0.67).	Provided the first comprehensive myocardial miRNA expression profile in sepsis, correlating alterations with post-mortem systemic inflammation.
